# Metastatic Choriocarcinoma of the Breast: A Rare Entity

**DOI:** 10.7759/cureus.22417

**Published:** 2022-02-20

**Authors:** Javaria Aleem, Pir Abdul Ahad Aziz Qureshi, Nida Babar, Anum Sultan, Anis Ur Rehman

**Affiliations:** 1 Department of Radiology, Shaukat Khanum Memorial Cancer Hospital and Research Centre, Lahore, PAK; 2 Department of Radiology, Syed Abdullah Shah Institute of Medical Sciences, Jamshoro, PAK; 3 Department of Pathology, Shaukat Khanum Memorial Cancer Hospital and Research Centre, Lahore, PAK

**Keywords:** metastatic metaplastic breast cancer, gestational trophoblastic disease, fdg pet-ct, breast choriocarcinoma, choriocarcinoma, breast cancer

## Abstract

Breast neoplasms are becoming more common in the last few years. Among these masses, metastasis to the breast is rare. Extra-gestational choriocarcinoma is extremely rare among breast neoplasms. We intend to present a case of a 30-year-old female with complaints of breast and axillary lumps. She had a history of a previously treated uterine trophoblastic tumor. Subsequently, she underwent a trucut biopsy of the breast mass and the axillary node, revealing metastatic choriocarcinoma.

## Introduction

The breast is an unusual site of extra-gestational choriocarcinoma. There are two varieties of breast choriocarcinoma: metastatic and breast cancer with choriocarcinoma features. In the literature, metastatic choriocarcinoma is documented in the lungs (80%), vagina (30%), pelvis (20%), liver, and brain (10%). Other rare sites include the spleen, kidney, and gastrointestinal tract. Metastasis to the breast is extremely rare with incidence ranging between 0.5% and 6.6% of all breast cancers. It is a high-grade disease with a poor survival rate. Breast choriocarcinoma presents as a well-circumscribed mass without spiculations, architectural distortion, and calcifications. They may be predominantly solid or has some cystic component.

This case report describes the history, physical examination, laboratory findings, imaging studies, and pathological findings of metastatic breast choriocarcinoma in a 30-year-old female. Most of the literature is about breast carcinoma with choriocarcinoma features [1].

## Case presentation

**Figure 1:**
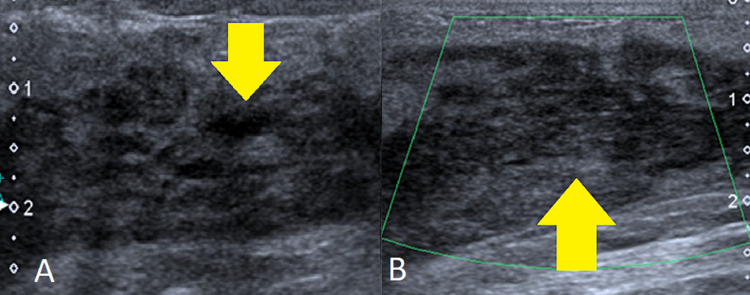
Ultrasound of the right breast Grayscale (A) and color Doppler (B) ultrasound images showing heterogenous infiltrative mass with internal cystic changes diffusely involving the outer quadrant and central part of the right breast (yellow arrows). No vascularity is seen on color Doppler images.

A 30-year-old female, a previously known case of choriocarcinoma of the uterus for which she underwent transabdominal hysterectomy and salpingo-oophorectomy in October 2019 followed by six cycles of chemotherapy, presented to the breast clinic of our institution with complaints of a lump in her right breast and right axilla and rising beta-hCG levels. After a general physical examination of the right breast mass and right axillary nodes, the patient was sent for ultrasound examination, which revealed a heterogenous infiltrative hypoechoic solid mass measuring about 7 cm with cystic foci diffusely involving the central and outer quadrants of the right breast from 8 o'clock to 12 o'clock position. No vascularity was seen on Doppler images (Figure 1).

**Figure 2:**
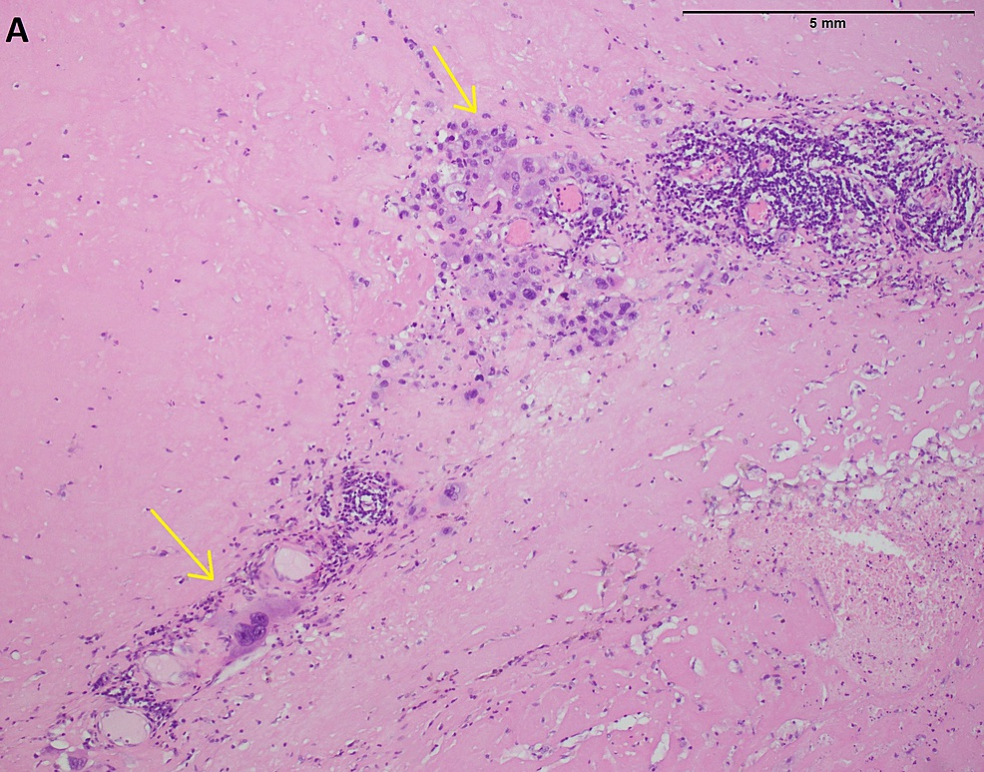
Histopathology A (high-power) image of breast parenchyma showing hyalinized breast parenchyma infiltrated by scattered tumor cells showing nuclear pleomorphism and multinucleation (yellow arrows).

The patient was subsequently referred for an ultrasound-guided biopsy. Histopathological examination of the mass revealed breast tissue with scattered solid sheets of atypical syncytiotrophoblast, cytotrophoblast, and intermediate trophoblast. On further immunohistochemical staining, it was positive for beta-hCG and negative for GCDFP, mammaglobin, and SALL4 (Figure 2).

**Figure 3:**
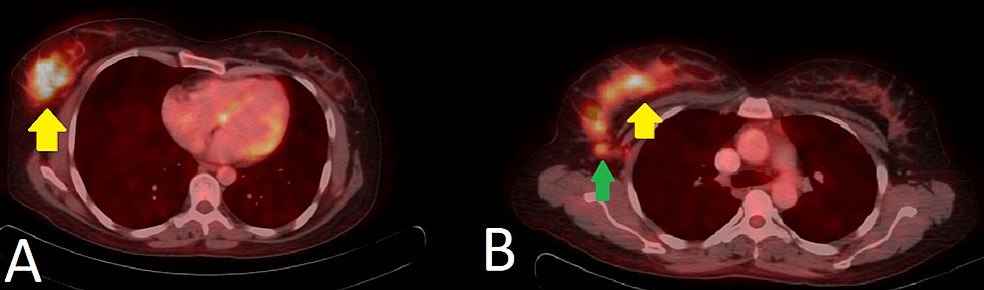
PET-CT scan (axial fused images) Hypermetabolic right breast mass (yellow arrows in A and B) with hypermetabolic axillary lymph nodes (green arrow in B).

Later, the patient underwent a PET-CT scan for a detailed metastatic workup, which revealed dense right breast parenchyma with heterogeneous mass showing hypermetabolic FDG uptake with SUVmax of 5.9 (background hepatic activity was 3.9 SUVmax). The mass extended up to the overlying skin with distinct fat planes. In addition, hypermetabolic right axillary subcentimeter nodes were also seen (Figure 3).

No other metastatic deposits or abnormal FDG uptake was seen at the vaginal stump (primary disease site). The case was then referred to the multidisciplinary tumor board (MDT), which recommended further chemotherapy. However, the patient opted for mastectomy. Therefore, a modified right breast radical mastectomy was performed. On monthly follow-up, the patient has declining beta-hCG levels.

## Discussion

Breast choriocarcinomas are very uncommon and can be divided into two varieties: breast cancer with choriocarcinomatous features (BCCF) and metastatic choriocarcinoma to the breast [2-4]. BCCF was first reported in 1981 by Saigo and Rosen in a 55-year-old female [1]. It is important to correctly differentiate between BCCF and metastatic breast choriocarcinoma because BCCF is an aggressive tumor with a poor prognosis [2].

The breast is an infrequent site of metastases from extra-mammary neoplasms with an incidence ranging from about 0.5% to 6.6% [2]. Choriocarcinoma usually metastasizes through the hematological pathway, and no organ is immune from choriocarcinomatous metastases [5]. Patients with metastatic breast choriocarcinoma usually present with a history of previous choriocarcinoma or a hydatidiform mole. It usually presents as a palpable lump in the breast with axillary lymphadenopathy and raised beta-hCG levels [2]. Additionally, many of these patients have a history of prior molar pregnancy, and patients develop breast lesions during or after pregnancy [5]. Interestingly, beta-hCG levels can also slightly raise in some common breast malignancies. The source of this hormone production in these cases is still unknown. However, high serum beta-hCG levels and the presence of beta-hCG antibodies are incredibly uncommon in usual breast carcinomas. This feature helps differentiate between breast metastasis and breast carcinoma with carcinomatous differentiation [2,6].

Radiologically, metastatic breast choriocarcinoma shares common radiological features with usual breast neoplasms. It may present as a high-density mass with obscured margins on mammograms without spiculations and calcifications. On ultrasound, it appears as a mixed solid and cystic hypoechoic mass with lobulated margins and a tendency of internal hemorrhage. PET scan is mandatory in these cases for the management to rule out multi-organ disease [6].

Most cases of breast carcinoma with choriocarcinoma features have an aggressive clinical course, and unfortunately, most of the reported cases in literature had a poor prognosis. Therefore, prompt diagnosis and early management are necessary. Detailed history, complete physical examination, imaging, histological and immunohistochemistry studies, and hormone serum analysis are necessary to differentiate between metastatic choriocarcinoma to the breast (extra-mammary origin) and breast carcinoma with choriocarcinoma features. A definite diagnosis is made through immunohistopathological examination.

## Conclusions

Metastatic breast choriocarcinomas are uncommon and are very aggressive lesions with a poor prognosis. However, chemotherapy can be given to improve the chances of survival. Additionally, it is also important to properly differentiate between BCCF and metastatic breast choriocarcinoma because BCCF has a more aggressive course as compared with metastatic breast choriocarcinoma.
